# Supplementary figures and images for: Forkhead transcription factor FKH-8 cooperates with RFX in the direct regulation of sensory cilia in Caenorhabditis elegans
Source: eLife. 2023 Jul 14;12:e89702. doi: 10.7554/eLife.89702 (PMC10393296; doi:10.7554/eLife.89702)

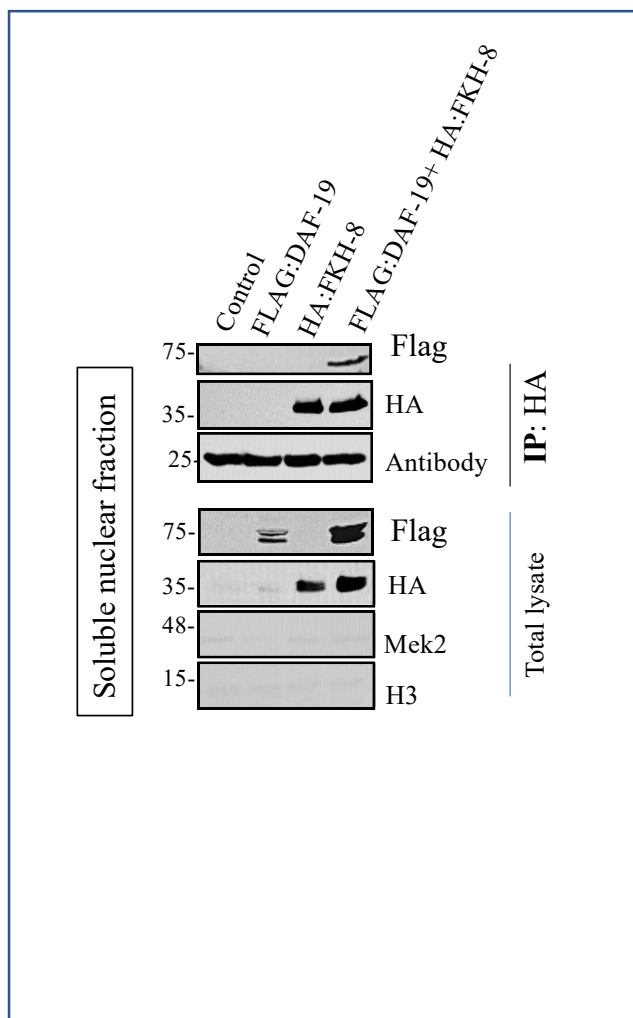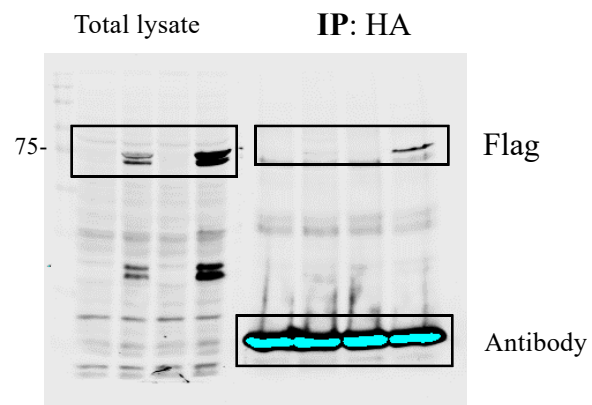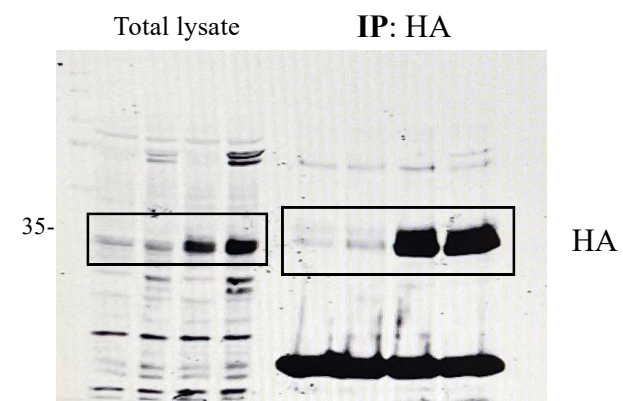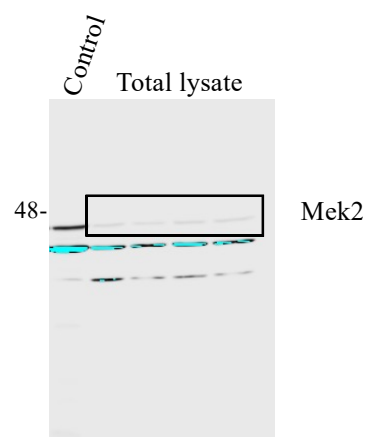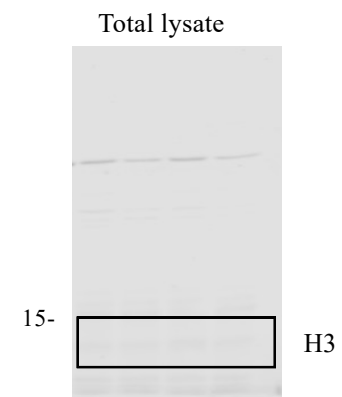

Supplement: Figure 2—source data 3. [file elife-89702-fig2-data3.zip › Figure2-source data 3/Original blot soluble nuclear.pdf]

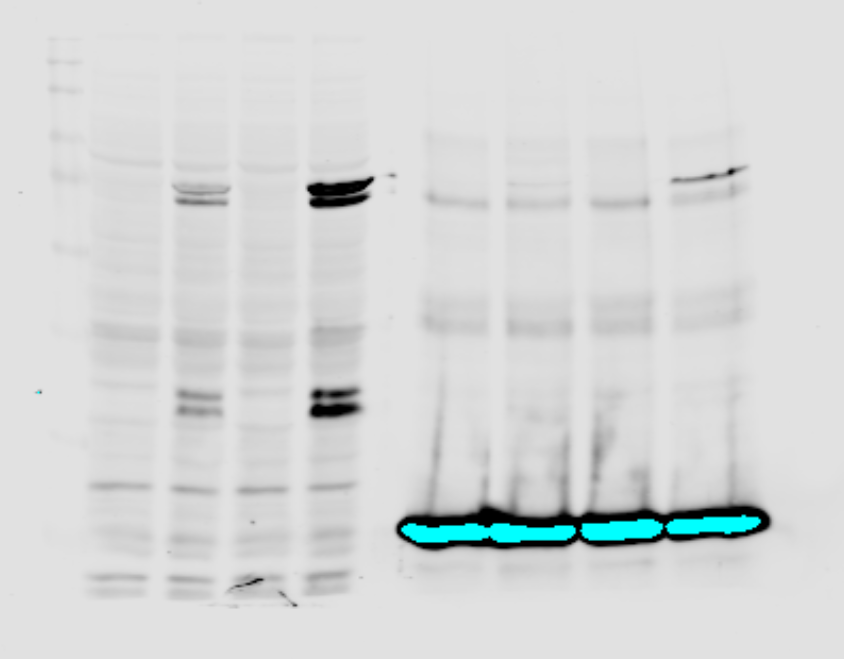

Supplement: Figure 2—source data 3. [file elife-89702-fig2-data3.zip › Figure2-source data 3/Flag.tif]

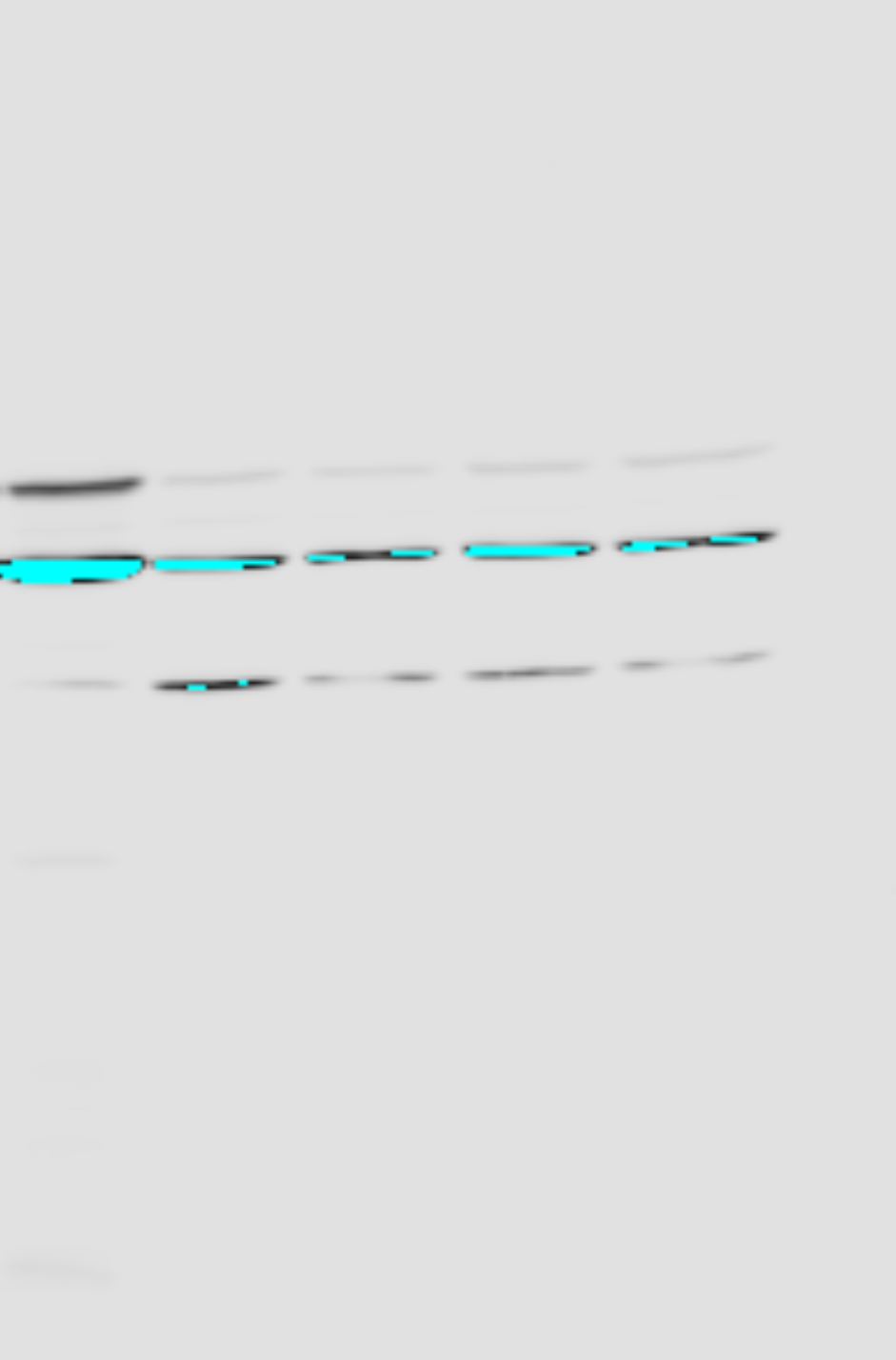

Supplement: Figure 2—source data 3. [file elife-89702-fig2-data3.zip › Figure2-source data 3/MEK2.tif]

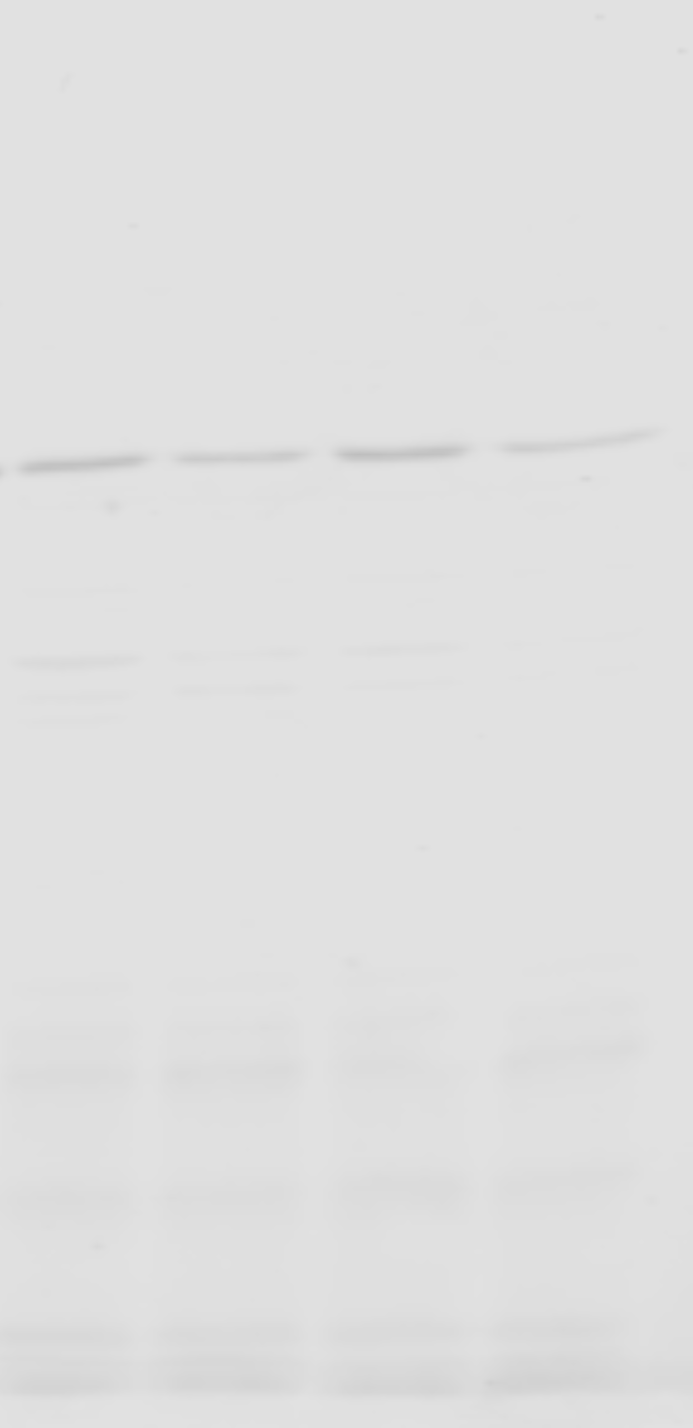

Supplement: Figure 2—source data 3. [file elife-89702-fig2-data3.zip › Figure2-source data 3/H3.tif]

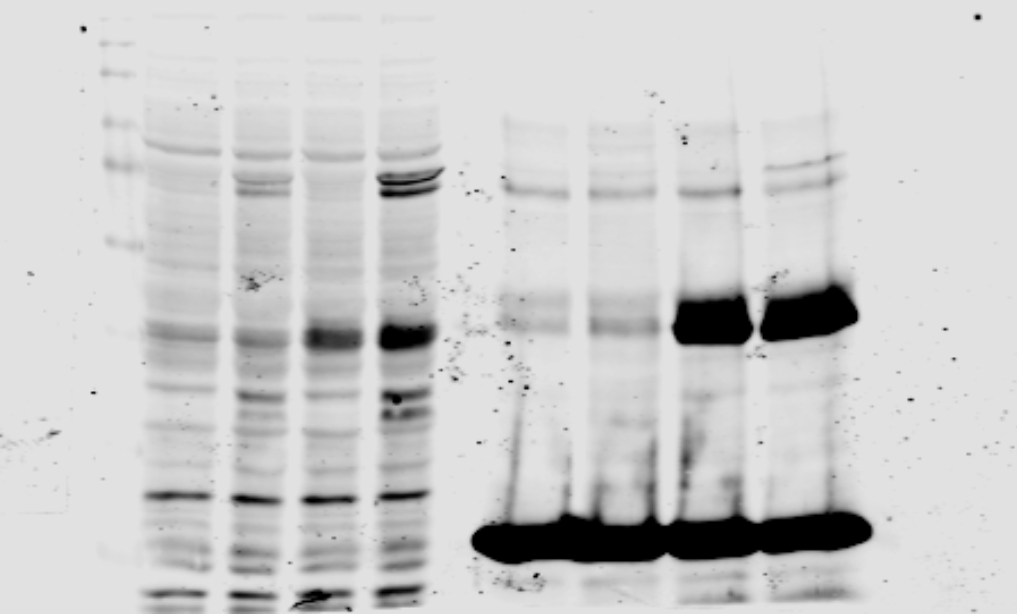

Supplement: Figure 2—source data 3. [file elife-89702-fig2-data3.zip › Figure2-source data 3/HA.tif]

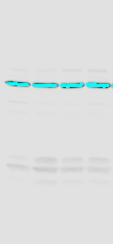

Supplement: Figure 2—figure supplement 2—source data 1. [file elife-89702-fig2-figsupp2-data1.zip › Figure 2 Figure Supplement 2-Source data 1/Mek2.png]

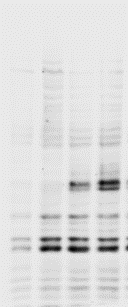

Supplement: Figure 2—figure supplement 2—source data 1. [file elife-89702-fig2-figsupp2-data1.zip › Figure 2 Figure Supplement 2-Source data 1/HA total.png]

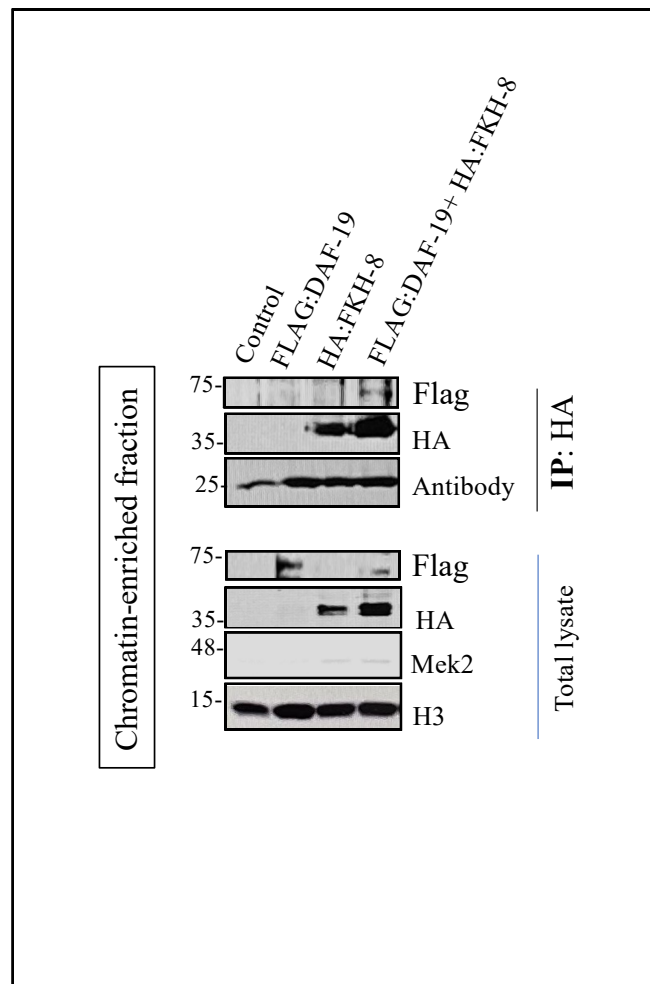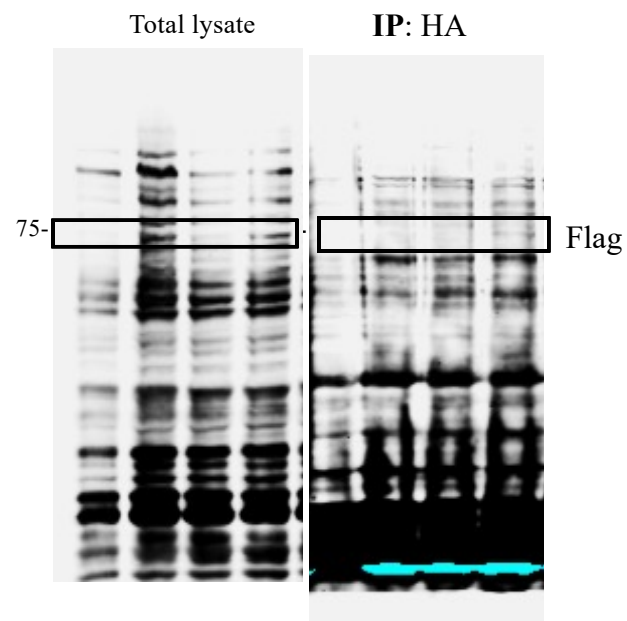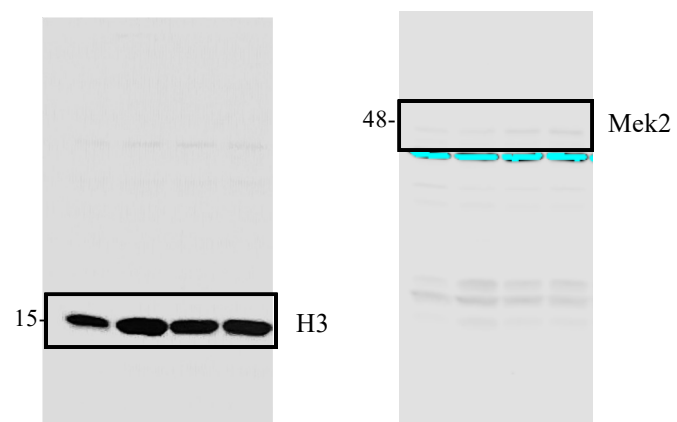

Supplement: Figure 2—figure supplement 2—source data 1. [file elife-89702-fig2-figsupp2-data1.zip › Figure 2 Figure Supplement 2-Source data 1/Original blot Chromatin.pdf]

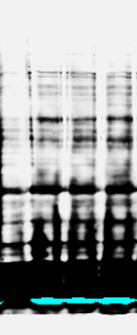

Supplement: Figure 2—figure supplement 2—source data 1. [file elife-89702-fig2-figsupp2-data1.zip › Figure 2 Figure Supplement 2-Source data 1/flag IP.png]

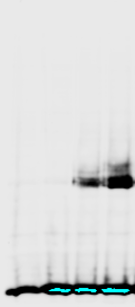

Supplement: Figure 2—figure supplement 2—source data 1. [file elife-89702-fig2-figsupp2-data1.zip › Figure 2 Figure Supplement 2-Source data 1/HA IP.png]

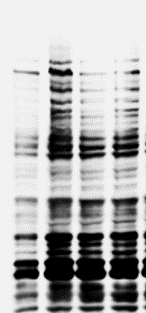

Supplement: Figure 2—figure supplement 2—source data 1. [file elife-89702-fig2-figsupp2-data1.zip › Figure 2 Figure Supplement 2-Source data 1/flag total.png]

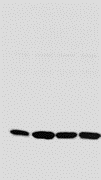

Supplement: Figure 2—figure supplement 2—source data 1. [file elife-89702-fig2-figsupp2-data1.zip › Figure 2 Figure Supplement 2-Source data 1/H3.png]
